# Comparative Proteomics of Extended-Spectrum Cephalosporin-Resistant *Neisseria gonorrhoeae* Isolates Demonstrates Altered Protein Synthesis, Metabolism, Substance Transport, and Membrane Permeability

**DOI:** 10.3389/fmicb.2020.00169

**Published:** 2020-02-19

**Authors:** Nannan Diao, Guoquan Yan, Yang Yang, Yuan Dong, Ying Wang, Weiming Gu

**Affiliations:** ^1^Department of Clinical Laboratory, Shanghai Skin Disease Hospital, Shanghai, China; ^2^Institutes of Biomedical Science, Fudan University, Shanghai, China; ^3^Shanghai Municipal Center for Disease Control and Prevention, Shanghai, China

**Keywords:** *Neisseria gonorrhoeae*, extended-spectrum cephalosporin resistance, comparative proteomics, iTRAQ, metabolism, membrane permeability, transportation, protein synthesis

## Abstract

*Neisseria gonorrhoeae* isolates exhibit resistance to extended-spectrum cephalosporins (ESCs), the last remaining option for first-line empirical monotherapy. Here, we investigated the proteomic profiles of *N. gonorrhoeae* clinical isolates with ESC-resistance to support exploration of the antimicrobial resistance mechanisms for *N. gonorrhoeae*. We used comparative iTRAQ quantitative proteomics to investigate differential protein expression of three ESC-resistant *N. gonorrhoeae* clinical isolates using *N. gonorrhoeae* ATCC49226 as a reference strain. The expression of 40 proteins was downregulated and expression of 56 proteins was upregulated in all three ESC-resistant *N. gonorrhoeae* isolates. Proteins with predicted function of translation, ribosomal structure and biogenesis, as well as components of the Type IV secretory systems, were significantly upregulated. Two differentially expressed proteins of ABC transporters were also reported by other teams in proteomics studies of *N. gonorrhoeae* isolates under antimicrobial stress conditions. Differentially expressed proteins are involved in energy production and metabolism of carbohydrates and amino acids. Our results indicated that amino acid and carbohydrate metabolism, cell membrane structure, interbacterial DNA transfer, and ribosome components might be involved in mediating ESC-resistance in *N. gonorrhoeae*. These findings facilitate a better understanding of the mechanisms of ESC-resistance in *N. gonorrhoeae* and provide useful information for identifying novel targets in the development of antimicrobials against *N. gonorrhoeae*.

## Introduction

Gonorrhea, caused by *Neisseria gonorrhoeae*, has been a major public global health concern (Newman et al., 2015; Unemo et al., 2017, 2019; Wi et al., 2017). Untreated and inappropriately treated gonorrhea can result in serious consequences on reproductive and neonatal health (Unemo and Shafer, 2014). There are no vaccines available for gonorrhea. *N. gonorrhoeae* has become resistant to multiple antimicrobials including the empirical first-line of treatment regimen, extended-spectrum cephalosporins (ESCs), such as ceftriaxone (CRO) and cefixime (CFM) (Bolan et al., 2012; Gu et al., 2014; Lewis, 2014; Yang et al., 2019).

Mutations have been associated with *N. gonorrhoeae* resistance or decreased susceptibility to CRO or CFM, such as mutations in *pen*A, *por*B, and *mtr*R genes (Liao et al., 2011; Unemo and Shafer, 2014). The *pen*A gene encodes penicillin-binding protein 2 (PBP2). Mutations in mosaic *pen*A is associated with ESC-resistance in *N. gonorrhoeae* (Spratt, 1988; Ameyama et al., 2002). The gene *por*B encodes a major porin PorB in the outer membrane, and mutations in PorB cause reduced influx of the ESC in *N. gonorrhoeae* (Olesky et al., 2006). At the level of protein expression, the multiple transferable resistance (MTR) system is one of the best studied systems in relation to ESC-resistance in *N. gonorrhoeae*. Mutations to the *mtr*R gene or the *mtr*R promoter sequence causes reduction or loss of MtrR repressive function on efflux pumps (Hagman and Shafer, 1995). This results in enhanced activity of the efflux complex and induces the expulsion of antibiotics from the cell (Golparian et al., 2014). However, the known resistance factors among gene/protein mutations cannot entirely explain the mechanism of ESC-resistance in *N. gonorrhoeae* (Ohnishi et al., 2011; Gong et al., 2016).

Comparative proteomics has been used to explore the mechanisms underlying antimicrobial resistance (AMR) in *N. gonorrhoeae*. Using two-dimensional gel electrophoresis and mass spectrometry (MS), Nabu et al. (2014) found that *N. gonorrhoeae* expressed higher levels of 50S ribosomal protein L7/L12 in response to spectinomycin (SPT) stimulation. In a comparative proteomics study, over 13 proteins were differentially expressed in response to ESC stimulation (Nabu et al., 2017). A proteomics study of the 2016 WHO *N. gonorrhoeae* reference strains with various profiles of AMR phenotypes and the *N. gonorrhoeae* strain FA6140 provided a reference proteomics databank for *N. gonorrhoeae* AMR research endeavors (El-Rami et al., 2019). Previous reports also revealed that expression of an outer membrane protein NGO1985 in *N. gonorrhoeae* was upregulated in response to antimicrobial stimulation (Zielke et al., 2014). The use of comparative proteomics to study mechanisms underlying *N. gonorrhoeae* AMR is still in its infancy (Baarda and Sikora, 2015). To our knowledge, the proteomics of clinical ESC-resistant *N. gonorrhoeae* isolates has not been explored thoroughly.

In this study, we utilized comparative proteomics to investigate the differential protein expressions in three ESC-resistant *N. gonorrhoeae* clinical isolates. Compared to ESC-susceptible reference strain *N. gonorrhoeae* ATCC49226, we found that the expression of 40 proteins was downregulated, and expression of 56 proteins was upregulated in all three ESC-resistant *N. gonorrhoeae* isolates. The differentially expressed proteins may play important roles in translation, ribosomal structure and biogenesis, Type IV secretion and transportation of molecules. Our results may facilitate identification of novel antimicrobial targets.

## Experimental Procedures

### Experimental Design and Statistical Rationale

Three ESC-resistant *N. gonorrhoeae* clinical isolates were identified and isolated in 2017 in Shanghai through the National Gonococcal Antimicrobial Susceptibility Surveillance Program of China. The three isolates were tentatively named *N. gonorrhoeae* SH40, SH41 and SH48. A laboratory *N. gonorrhoeae* strain ATCC49226 was used as a reference. The antimicrobial susceptibility of the four isolates was assessed for CRO, CFM, SPT, ciprofloxacin (CIP), penicillin (PEN), tetracycline (TET), and azithromycin (AZM) using the agar dilution method (Clinical and Laboratory Standard Institute [CLSI], 2014). Beta-lactamase producing *N. gonorrhoeae* (PPNG) isolates were determined using the nitrocefin test (Gu et al., 2014). Plasmid-mediated tetracycline-resistant *N. gonorrhoeae* (TRNG) isolates were defined as those having a TET MIC of ≥16.0 mg/L (Gu et al., 2014). This study was approved by the Ethics Committee of the Shanghai Skin Disease Hospital.

Tandem mass spectra were analyzed using the PEAKS Studio version 8.5 (Bioinformatics Solutions Inc., Waterloo, ON, Canada) to search the Uniprot_NeisseriaGonorrhoeae (201805, 19434 entries) database, assuming that trypsin is the digestion enzyme and that a maximum of two missed cleavages is permitted. The PEAKS database was searched with a fragment ion mass tolerance of 0.05 Da and a parent ion tolerance of 10 ppm. Carbamidomethylation (C) and iTRAQ 4plex (K, N-term) were specified as the fixed modification. Oxidation (M), Deamidation (NQ), and Acetylation (Protein N-term) were specified as the variable modifications. Peptides were filtered with a 1% false discovery rate (FDR) and were required to be a unique peptide. PEAKS Q was used for peptide and protein abundance calculation. Quantification results of proteins were validated using the PEAKS Q Method. Log_10_ (protein abundance) was used for significance testing and calculation and the information of the peptides and the proteins (Supplementary Data Sheet S1). Normalization was performed by calculating a global ratio from the total intensity of all labels in all quantifiable peptides. Normalized abundance was calculated from the raw abundance divided by the normalization factor. Differentially expressed proteins were filtered if their fold change (FC) was over 1.5 or below −1.5 as compared to ATCC49226 and contained at least 2 unique peptides (*P* < 0.01) (Baarda et al., 2019; El-Rami et al., 2019).

### Growth of *Neisseria gonorrhoeae* Isolates and Preparation of Protein Extract for Proteomics Analysis

*Neisseria gonorrhoeae* cells were cultivated on GC agar plates and incubated for 20 h in 5% CO_2_ at 37°C (Wu et al., 2010; Nabu et al., 2017; El-Rami et al., 2019). Subsequently, cells were harvested in 0.1 M phosphate-buffered solution (PBS), pH 7.4. Bacterial suspension was centrifuged at 10,000 × *g* at 4°C for 20 min. Cell pellets were washed with PBS three times. Pellets were re-suspended in 1 mL lysis buffer [7 M urea, 2% sodium dodecylsulfate (SDS), and 1× Protease Inhibitor (Cocktail Roche Ltd., Basel, Switzerland)]. Bacterial suspension was subjected to sonication (300 W) on ice. Cell lysate was centrifuged at 13,000 rpm for 10 min at 4°C. The supernatant was transferred to fresh tubes.

Protein concentrations in the supernatant were determined using the bicinchoninic acid (BCA) assay. The protein solutions were diluted to 100 μg per 100 μL with 100 mM triethylammonium bicarbonate (TEAB). Next, 5 μL 200 mM dichlorodiphenyltrichloroethane (DTT) was added to the solutions. Protein samples were incubated at 55°C for 1 h, and then 5 μL of 375 mM iodoacetamide was added. The samples were further incubated for 30 min at room temperature with protection from light. Proteins were precipitated with ice-cold acetone and were re-dissolved in 100 μL of 100 mM TEAB.

### 4-plex iTRAQ Proteomics

First, proteins were digested with sequence-grade modified trypsin (Promega, Madison, WI, United States). The resultant peptide mixtures were labeled using chemicals from the iTRAQ 4plex reagent kits. Tryptic peptides of *N. gonorrhoeae* ATCC49226, SH40, SH41 and SH48 were labeled with 114, 115, 116 and 117 iTRAQ tags, respectively. Peptides were then acidified using 150 μL of 1% formic acid. Then the four samples were mixed and desalted using a C18 SPE column (Sep-Pak C18, Waters, Milford, MA, United States) and dried in a vacuum concentrator.

Each of the four samples was subjected to chromatography studies in triplicate. To preliminarily separate peptides, the peptide mixture was re-dissolved in the buffer A (10 mM ammonium formate, pH 10.0 adjusted with NH_4_OH) and fractionated by high pH separation using a reverse phase column (BEH C18 column, 2.1 mm × 150 mm, 1.7 μm, 300 Å, Waters Corporation, Milford, MA, United States) and an Aquity UPLC system (Waters Corporation, Milford, MA, United States). High pH separation was performed using a linear gradient of 0% buffer B to 45% buffer B in 35 min (Buffer B: 10 mM ammonium formate in 90% ACN, pH 10.0 adjusted with NH_4_OH). The column flow rate was maintained at 250 μL/min, and the column temperature was maintained at 45°C. Twelve fractions were collected, and each fraction was dried in a vacuum concentrator for low pH Nano-HPLC-MS/MS analysis.

To conduct low pH nano-HPLC-MS/MS analysis, each fraction was re-suspended with 40 μL solvent C (water with 0.1% formic acid) and solvent D (ACN with 0.1% formic acid). The mixture was then separated by nanoLC and analyzed by on-line electrospray tandem MS. The experiments were performed on a Nano Aquity UPLC system (Waters Corporation, Milford, MA, United States) connected to a quadrupole-Orbitrap mass spectrometer (Q-Exactive; Thermo Fisher Scientific, Bremen, Germany) equipped with an online nano-electrospray ion source. An amount of 4 μL peptide sample was loaded onto the trap column (Thermo Scientific Acclaim PepMap C18, 100 μm × 2 cm) at a flow rate of 10 μL/min for 3 min. The eluents were subsequently separated on an analytical column (Acclaim PepMap C18, 75 μm × 25 cm) with a linear gradient of 5% solvent D to 30% solvent D in 110 min. The column was cleaned and then was re-equilibrated with initial conditions for 10 min. The column flow rate was maintained at 300 μL/min, and the column temperature was maintained at 45°C. The electrospray voltage of 2.0 kV versus the inlet of the mass spectrometer was used. The spectrometer analysis was conducted in triplicates for the peptide sample.

The Q-Exactive mass spectrometer was operated in the data-dependent mode to switch automatically between MS and MS/MS acquisition. A survey of full-scan MS spectra (m/z 350–1600) was acquired with a mass resolution of 7 × 10^4^, followed by 10 sequential high-energy collisional dissociation (HCD) MS/MS scans with a resolution of 1.75 × 10^4^. Ions with charge states 2+, 3+, and 4+ were fragmented with an isolation window of 2 m/z. For MS scans, the AGC target was set to 5 × 10^5^, and the maximum injection time was 50 ms. For MS/MS scans, the AGC target was set to 2 × 10^5^, and the maximum injection time was 100 ms. In all cases, one microscan was recorded using dynamic exclusion of 30 s. MS/MS fixed first mass was set at 100.

### Bioinformatics Analysis

Whole protein sequences were analyzed using the BlastP program. A combination of bioinformatics tools including PSORTb 3.0.2, CELLO 2.5, and SOSUIGramN were used to analyze the subcellular distribution of the differential expressed proteins. A majority-votes strategy was chosen for the cellular assignment of proteins. When the subcellular distributions search for a protein using the three tools were inconsistent, that protein was classified as “unknown.” Furthermore, the predicted amino acid sequences of proteins were analyzed for the presence and location of signal peptides cleavage sites using the SignalP-5.0 program. Prediction of transmembrane helices of proteins was performed by the TMHMM 2.0 program. Analysis of cluster of orthologous groups (COG) was based on the phylogenetic classification of proteins encoded in complete genomes project (NCBI)^1^. The gene ontology (GO) database was used to explore functional annotations. Statistically altered functions of differentially expressed proteins were calculated by Fisher’s exact test in BLAST2GO version 4. Pathway analysis was conducted using the KOBAS program^2^. To determine the possible protein-protein interactions (PPI) and the network analysis for protein groups, the Cytoscape v3.7 and STRING v10.5 programs were used. Proteins with network nodes >2 were selected. The “network analyzers” were used to analyze the topological properties of the network. The MCODE and cytoHubba APP programs were used to define hub proteins.

### Quantitative Reverse Transcription Polymerase Chain Reaction (qRT-PCR)

A total of 28 proteins with the highest fold changes were selected from the 96 significant differentially expressed proteins for confirmation of their RNA expression level using the qRT-PCR assays with specific primers (Supplementary Table S1). Total RNA was extracted from *N. gonorrhoeae* cells using TRIzol reagent (Thermo Fisher Scientific, MA, United States) and PureLink RNA Mini Kit (Thermo Fisher Scientific, MA, United States) according to the manufacturer’s instructions. Then RNA was reverse transcribed into cDNA with Revert Aid Premium Reverse Transcriptase (Thermo Scientific^TM^ EP0733). The reverse transcription product was amplified and detected with SYBR^®^ Premix Ex Taq^TM^ (Takara Bio Inc., Otsu, Japan) in an ABI StepOnePlus^TM^ Real-Time PCR system (Applied Biosystems, Foster City, CA, United States). qRT-PCR for each sample was conducted in triplicate. Data was analyzed according to relative gene expression using the 2^–ΔΔCt^ method.

### Statistical Analysis

Statistical analysis was performed using GraphPad Prism version 7.0 (GraphPad Software, Inc., La Jolla, CA, United States). *P* values of less than 0.05 (*P* < 0.05) were considered statistically significant.

## Results

### Antimicrobial Susceptibility Profiles of *Neisseria gonorrhoeae* Isolates

The minimum inhibitory concentrations (MICs) of antimicrobials were determined for the three *N. gonorrhoeae* isolates, SH40, SH41 and SH48. *N. gonorrhoeae* SH40, SH41 and SH48 had CRO MICs ≥1.0 mg/L and CFM MICs ≥1.0 mg/L. These three *N. gonorrhoeae* isolates were resistant to CIP and PEN and were susceptible to AZM and SPT. *N. gonorrhoeae* SH40 and SH48 were resistant to TET, while *N. gonorrhoeae* SH41 was susceptible to TET. All the three isolates were non-PPNG and non-TRNG isolates. The antimicrobial susceptibility profile of *N. gonorrhoeae* ATCC49226 has been characterized by others (Nabu et al., 2014, 2017), and we confirmed that it is susceptible to CRO (MIC = 0.015) and CFM (MIC < 0.004).

### Differential Protein Expression in *Neisseria gonorrhoeae* Isolates

**FIGURE 1:**
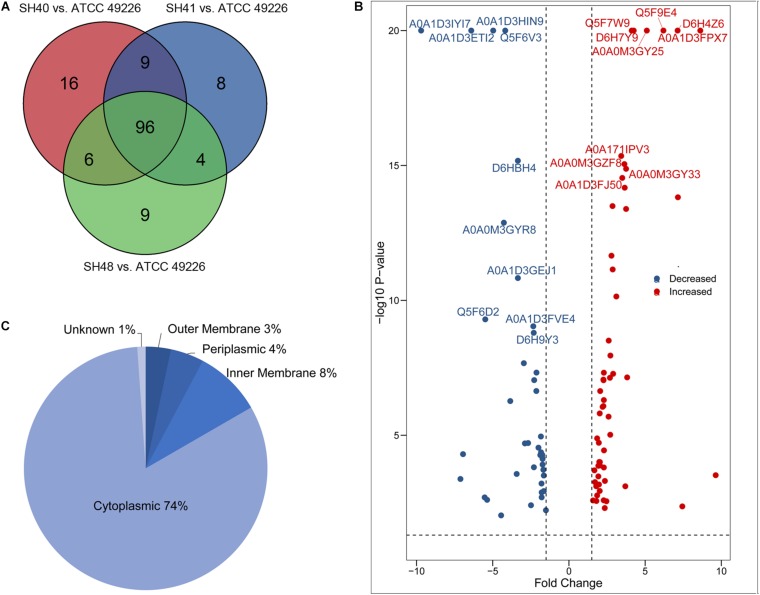
Proteome profiling in *Neisseria gonorrhoeae* ATCC49226 and three ESC-resistant *N. gonorrhoeae* clinical isolates. **(A)** Venn diagrams illustrating the distribution of differentially expressed proteins of 3 clinical isolates compared to the reference strain ATCC49226. Ninety-six differentially expressed proteins common to the 3 clinical isolates were revealed, while a total of 127, 117, and 115 differentially expressed proteins were identified in *N. gonorrhoeae* isolate SH40, SH41 and SH48, respectively. **(B)** Volcano plot of protein abundance differences. The top 10 significantly differentially expressed proteins with lowest *P* value were showed. **(C)** Predicted cellular localizations of 96 differentially expressed proteins.

**FIGURE 2:**
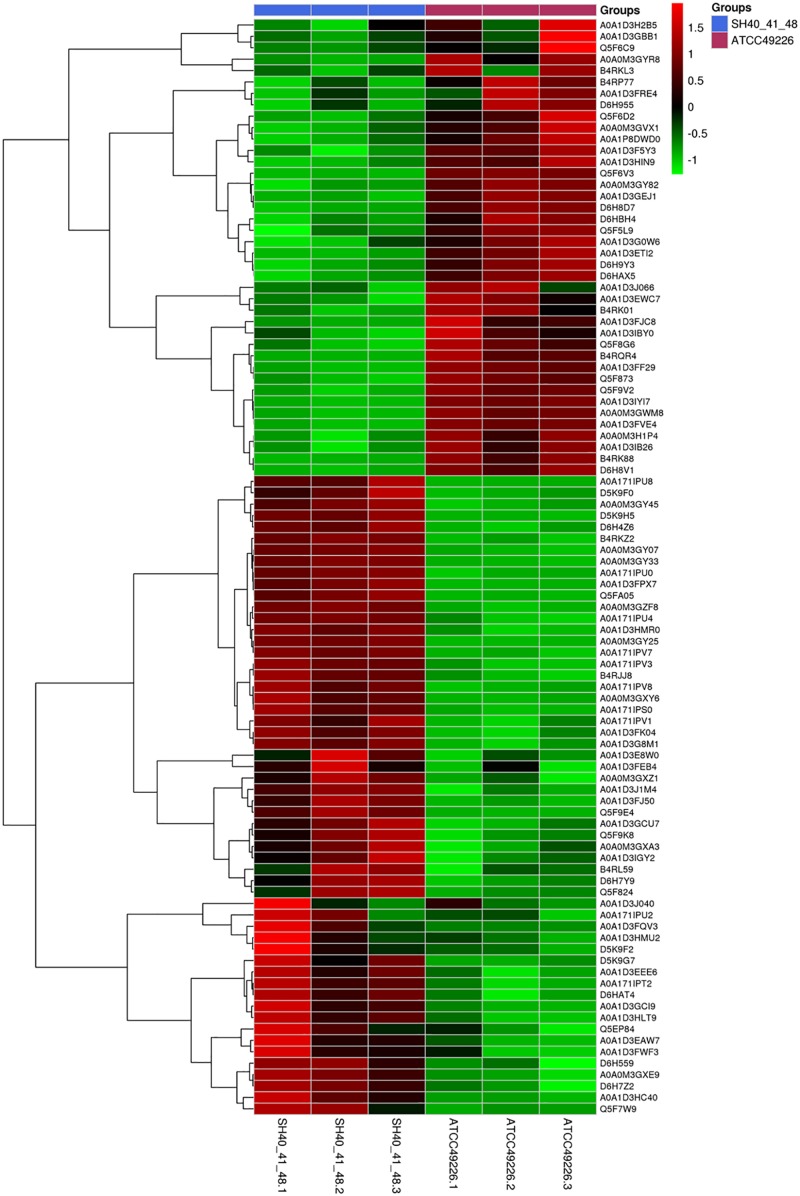
Heirarchical clustering analysis of 96 differentially expressed proteins in *N. gonorrhoeae* isolates. Red and green squares indicate higher and lower abundances compared with the average expression, respectively.

A total of 1664 proteins were detected. Over 100 proteins exhibited differential expression in the three *N. gonorrhoeae* isolates, i.e., 127 proteins for *N. gonorrhoeae* SH40, 117 proteins for *N. gonorrhoeae* SH41, and 115 proteins for *N. gonorrhoeae* SH48 when compared to the expression profiles of *N. gonorrhoeae* ATCC49226 (Figure 1A). Further, 96 proteins exhibited altered expression levels in all three *N. gonorrhoeae* isolates by at least 1.5-folds in comparison to *N. gonorrhoeae* ATCC49226 (unique peptides ≥ 2; *P* < 0.01) (Figure 1A and Table 1). Among the significantly differentially expressed proteins, 40 had decreased expression while 56 had increased expression. The top 10 significantly differentially down- or up-regulated proteins with lowest *P* values are also shown (Figure 1B). Prediction was made for the subcellular distribution, signal peptides cleavage sites, and transmembrane helices (TMHs) of the differentially expressed proteins (Table 1). The majority of these 96 differentially expressed proteins localized in the cytoplasm (71/96), and the remainder localized to the outer membrane (3/96), periplasm (4/96), inner membrane (8/96), and unknown locations (10/96) (Figure 1C). Moreover, hierarchical cluster analysis (HCA) showed that the proteomic profiles of the three isolates clustered distinctly from *N. gonorrhoeae* ATCC49226 (Figure 2).

**TABLE 1:** List of differentially expressed proteins in 3 ESC-resistant *N. gonorrhoeae* isolates.

**UniProt entry**	**Gene name^a^**	**Protein description**	**COG^#^**	**Localization**	**TMHs^b^**	**SignalP^c^**	FC*
A0A1D3GBB1	*WHOM_00054C*	PilC2	NW	Outer membrane	1	Y	−4.45
B4RQR4	*NGK_2270*	Adhesin MafA	–		1	Y	−5.37
D5K9H5	*NCTC10931_02413*	TrbJ	X		1	Y	7.15
A0A171IPU2	*NGTW08_p0027*	TrbG	U	Periplasmic	0	Y	2.28
A0A1D3G8M1	*WHOG_00181C*	ABC transporter substrate–binding protein	P		0	Y	1.85
A0A1D3HMU2	*WHOL_PCO00017*	Conjugal transfer protein TrbM	–		0	Y	2.35
A0A1D3J040	*fimT*	Fimbrial protein FimT	NW		1	N	2.46
A0A0M3GWM8	*M736_07665*	Biopolymer transporter ExbD	U	Inner membrane	1	N	−1.8
B4RP77	*NGK_1737*	NAD(P) transhydrogenase subunit beta	C		9	N	−1.68
A0A0M3GXA3	*M736_06455*	Phosphate transporter	P		11	N	1.71
A0A171IPU4	*NGTW08_p0029*	TrbE	U		2	N	2.02
A0A171IPV7	*NGTW08_p0042*	TraM	–		1	N	3.66
A0A1D3E8W0	*WHOL_00301*	Membrane protein	–		2	N	2.03
A0A1D3FJ50	*WHOL_01550*	Conjugal transfer protein TrbF	U		1	N	3.5
A0A1D3FK04	*NA*	NA	U		3	N	2.26
A0A0M3GVX1	*M736_08600*	Uncharacterized protein	–	Cytoplasmic	0	N	−2
A0A0M3GY82	*aspA*	Aspartate ammonia–lyase	E		0	N	−1.73
A0A0M3GYR8	*ileS*	Isoleucine-tRNA ligase	J		0	N	−4.27
A0A0M3H1P4	*M736_09830*	Phosphoglycolate phosphatase	C		0	N	−2.14
A0A1D3ETI2	*tsf*	Elongation factor Ts	J		0	N	−6.41
A0A1D3EWC7	*WHOL_00865*	PhnO–like protein	J		0	N	−1.83
A0A1D3F5Y3	*dmlR_2*	LysR family transcriptional regulator	K		0	N	−1.65
A0A1D3FF29	*NA*	NA	EH		0	N	−1.84
A0A1D3FJC8	*WHOG_00311*	Uncharacterized protein	–		0	N	−5.53
A0A1D3FRE4	*glpR*	DeoR family transcriptional regulator	KG		0	N	−2.49
A0A1D3FVE4	*ssuB*	ABC transporter ATP–binding protein	P		0	N	−2.34
A0A1D3G0W6	*NA*	NA	–		0	N	−1.79
A0A1D3GEJ1	*WHOF_01391*	Predicted ATP–binding protein involved in virulence	L		0	N	−3.35
A0A1D3H2B5	*vacB*	Ribonuclease R	K		0	N	−2.3
A0A1D3HIN9	*NCTC10931_00949*	Phage associated protein	X		0	N	−4.97
A0A1D3IB26	*NCTC10931_02268*	Aminotransferase	E		0	N	−1.74
A0A1D3IBY0	*NCTC10931_00390*	Type II restriction endonuclease *Nla*IV	–		0	N	−2.68
A0A1D3IYI7	*WHOF_00049C*	Uncharacterized protein	–		0	N	−9.7
A0A1D3J066	*WHOF_00223*	Glycosyltransferase PglE	G		2	N	−3.85
A0A1P8DWD0	*mtrR*	MtrR	K		0	N	−2.96
B4RK01	*NGK_0461*	PTS system, nitrogen regulatory IIA protein	GT		0	N	−1.79
B4RK88	*NGK_0548*	Putative oxidoreductase, NAD(P)H–flavin	C		0	N	−7.12
D6H8D7	*NGMG_01320*	Uncharacterized protein	C		0	N	−1.65
D6H8V1	*NGMG_01484*	Succinate semialdehyde dehydrogenase	C		0	N	−2.28
D6H955	*NGMG_00060*	Type I restriction–modification system specificity determinant	V		0	N	−3.43
D6H9Y3	*asd*	Aspartate–semialdehyde dehydrogenase	E		0	N	−2.31
D6HAX5	*NGMG_00527*	Myo–inositol–1(Or 4)–monophosphatase	G		0	N	−1.88
D6HBH4	*NGMG_02139*	3–hydroxyacid dehydrogenase	I		0	N	−3.35
Q5F5L9	*dksA*	RNA polymerase–binding transcription factor DksA	J		0	N	−1.5
Q5F6C9	*NGO_1630*	Repressor	X		0	N	−6.95
Q5F6D2	*NGO_1627*	Uncharacterized protein	K		0	N	−5.49
Q5F6V3	*adhP*	Ethanol–active dehydrogenase/acetaldehyde–active reductase	G		0	N	−4.18
Q5F873	*gltA*	Citrate synthase	C		0	N	−2.13
Q5F8G6	*ilvD*	Dihydroxy–acid dehydratase	EG		0	N	−1.73
Q5F9V2	*NGO_0286*	Uncharacterized protein	S		0	N	−1.75
A0A0M3GXE9	*M736_02645*	Conjugal transfer protein TrbB	UW		0	N	2.36
A0A0M3GXY6	*M736_02455*	Phosphoribosyltransferase	F		0	N	2.69
A0A0M3GXZ1	*M736_02480*	Conjugal transfer protein TraJ	–		0	N	1.8
A0A0M3GY07	*tsf*	Elongation factor Ts, EF–Ts	J		0	N	3.75
A0A0M3GY25	*M736_02655*	Single–stranded DNA–binding protein	L		0	N	5.11
A0A0M3GY33	*M736_02680*	Peptide transporter	D		0	N	3.75
A0A0M3GY45	*M736_02210*	Cytosine–specific methyltransferase	L		0	N	2.26
A0A0M3GZF8	*M736_02560*	Toxin	R		0	N	3.64
A0A171IPS0	*NGTW08_p0005*	TraC	L		0	N	2.86
A0A171IPT2	*NGTW08_p0017*	MarR	–		0	N	1.97
A0A171IPU8	*NGTW08_p0033*	Uncharacterized protein	K		0	N	3.82
A0A171IPV8	*NGTW08_p0043*	TraL	N		0	N	2.87
A0A1D3EAW7	*yhaV*	Toxin YhaV	–		0	N	2.9
A0A1D3FEB4	*WHOG_00308*	Uncharacterized protein	–		0	N	2.02
A0A1D3FQV3	*infB*	Translation initiation factor IF–2	J		0	N	9.63
A0A1D3FWF3	*WHOV_00938*	Acetyltransferase	J		0	N	1.79
A0A1D3GCI9	*NCTC10931_01742*	Uncharacterized conserved small protein	R		0	N	3.11
A0A1D3GCU7	*WHOG_01258*	Uncharacterized protein	–		0	N	2.29
A0A1D3HC40	*rsmI_1*	Ribosomal RNA small subunit methyltransferase I	J		0	N	2.21
A0A1D3HLT9	*topB*	DNA topoisomerase 3	L		0	N	2.29
A0A1D3HMR0	*NCTC10931_02384*	Uncharacterized protein	–		0	N	2.3
A0A1D3IGY2	*WHOP_01429C*	Uncharacterized protein conserved in bacteria	S		0	N	1.99
A0A1D3J1M4	*rpsF*	30S ribosomal protein S6	J		0	N	2.72
B4RJJ8	*NGK_0308*	Restriction endonuclease R.NgoMIII	L		0	N	1.96
B4RKZ2	*NGK_0802*	ADP–heptose—-LPS heptosyltransferase II	M		0	N	1.58
D5K9F0	*NCTC10931_02421*	Epsilon_1 antitoxin	–		0	N	3.7
D5K9F2	*NCTC10931_02377*	Epsilon_3 antitoxin	–		0	N	2.29
D5K9G7	*NCTC10931_02420*	Zeta_1 toxin	–		0	N	2.71
D6H4Z6	*NGMG_01890*	16S RNA methyltransferase	J		0	N	7.13
D6H7Z2	*NGMG_01175*	Uncharacterized protein	KV		0	N	2.61
D6HAT4	*NGMG_00403*	Riboflavin biosynthesis protein RibD	H		0	N	2.6
Q5EP84	*yecA*	YecA	S		0	N	1.85
Q5F7W9	*clpB*	Chaperone protein ClpB	O		0	N	4.25
Q5F824	*ackA*	Acetate kinase	C		0	N	7.44
Q5F9K8	*NGO_0385*	Delta–aminolevulinic acid dehydratase	H		0	N	1.67
Q5FA05	*NGO_0229*	Uncharacterized protein	–		0	N	2.27
B4RKL3	*NGK_0673*	Putative phage associated protein	–	Unknown	0	N	−2.89
A0A171IPU0	*NGTW08_p0025*	TrbI	U		1	N	2.79
A0A171IPV1	*NGTW08_p0036*	KorC	–		0	N	1.93
A0A171IPV3	*NGTW08_p0038*	IncC2	N		0	N	3.43
A0A1D3EEE6	*sodB*	Superoxide dismutase	P		0	N	2.05
A0A1D3FPX7	*vapD_1*	VapD	S		0	N	8.62
B4RL59	*rpmE2*	50S ribosomal protein L31 type B	J		0	N	1.93
D6H559	*NGMG_01953*	Uncharacterized protein	–		0	Y	2.03
D6H7Y9	*NGMG_01172*	Uncharacterized protein	–		0	Y	4.12
Q5F9E4	*NGO_0454*	Pilus assembly protein PilW	NW		1	N	6.2

^*a*^Gene name from Uniprot. ^*b*^Number of predicted transmembrane helix (TMHs) using TMHMM. ^*c*^Prediction of signal peptide cleavage site using SignalP. Y, peptide cleavage site was predicted; N, no peptide cleavage site was predicted. NA, no annotation; –, decrease; +, increase. *Calculation of fold change (FC): Differentially expressed proteins were filtered if their fold change (FC) was over 1.5 or below −1.5 relative to ATCC49226 and contained at least 2 unique peptides (*P* < 0.01). ^#^COG represents clusters of orthologous groups, the abbreviations are annotated in Figure 3.

### Differentially Expressed Proteins Are Associated With Basal Metabolism, Ribosomal Structure, and Vesicular Transport in *Neisseria gonorrhoeae*

**FIGURE 3:**
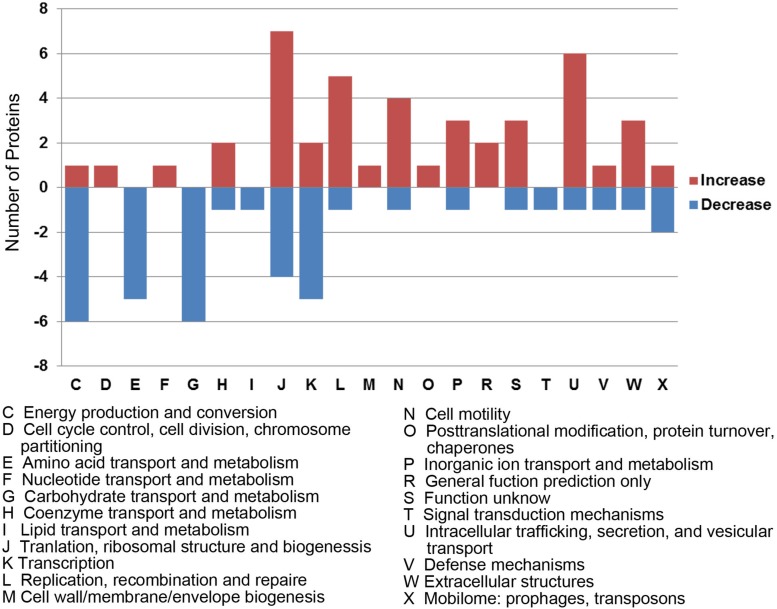
Functional classification of differentially expressed proteins according to clusters of orthologous groups (COGs). Histogram illustrating the COG functional categories and the associated protein number. –, proteins with decreased expression.

To better understand the functions and relationships of the 96 differentially expressed proteins, we performed COG and GO enrichment analysis. The highest scoring category in COG analysis was “Translation, ribosomal structure and biogenesis” (J category), which included 11 differentially expressed proteins (7 upregulated and 4 downregulated) (Figure 3). COG analysis also identified metabolism categories, such as “Energy production and conversion” (C category), “Amino acid transport and metabolism” (E category), and “Carbohydrate transport and metabolism” (G category). The differentially expressed proteins common to all three strains involved in metabolism categories were generally downregulated. In addition, a protein associated with “lipid transport and metabolism” (I category) was found to be downregulated (D6HBH4, 3-hydroxyacid dehydrogenase). Other function categories were “Intracellular trafficking, secretion, and vesicular transport” (U category) and “Extracellular structures” (W category). Proteins of categories U and W are associated with the Type IV secretory pathway (Table 2). Proteins associated with transcriptional regulation were observed. We found that 5 out of 7 proteins were downregulated in the “Transcription” (K category), while 5 out of 6 proteins were upregulated in “Replication, recombination, and repair” (L category). Another significantly upregulated protein was B4RKZ2 (ADP-heptose-LPS heptosyltransferase II) belonging to the class of “Cell wall/membrane/envelope biogenesis”. Differential expression of MtrR (A0A1P8DWD0) and a predicted membrane protein (A0A1D3E8W0) was detected.

**TABLE 2:** Differentially expressed proteins associated with Type IV secretory pathway.

**UniProt entry**	**Gene name**	**COG term**	**Protein name**	**FC**
A0A1D3GBB1	*WHOM_00054C*	Tfp pilus assembly protein, tip-associated adhesin PilY1	PilC2	−4.45
A0A0M3GXE9	*M736_02645*	Pilus assembly protein, ATPase of CpaF family	TrbB	2.36
A0A0M3GXZ1	*M736_02480*	–	TraJ	1.8
A0A171IPS0	*NGTW08_p0005*	Antirestriction protein ArdC	TraC	2.86
A0A171IPU0	*NGTW08_p0025*	Type IV secretory pathway, VirB10 components	TrbI	2.79
A0A171IPU2	*NGTW08_p0027*	Type IV secretory pathway, VirB9 components	TrbG	2.28
A0A171IPU4	*NGTW08_p0029*	Type IV secretory pathway, VirB4 component	TrbE	2.02
A0A171IPV3	*NGTW08_p0036*	Cellulose biosynthesis protein BcsQ	IncC2	3.43
A0A171IPV7	*NGTW08_p0042*	–	TraM	3.43
A0A171IPV8	*NGTW08_p0043*	Cellulose biosynthesis protein BcsQ	TraL	3.66
A0A1D3FJ50	*WHOL_PCO00023*	Type IV secretory pathway, TrbF components	TrbF	3.5
A0A1D3FK04	*NA*	Type IV secretory pathway, VirD4 component, TraG/TraD family ATPase	NA	2.26
A0A1D3HMU2	*WHOL_PCO00017*	–	TrbM	2.35
A0A1D3J040	*fimT*	Tfp pilus assembly protein FimT	FimT	2.46
D5K9H5	*NCTC10931_02413*	Conjugal transfer/entry exclusion protein	TrbJ	7.15
Q5F9E4	*NGO_0454*	Tfp pilus assembly protein PilW	PilW	6.2

*FC, fold change; NA, not available; −, not available.*

**FIGURE 4:**
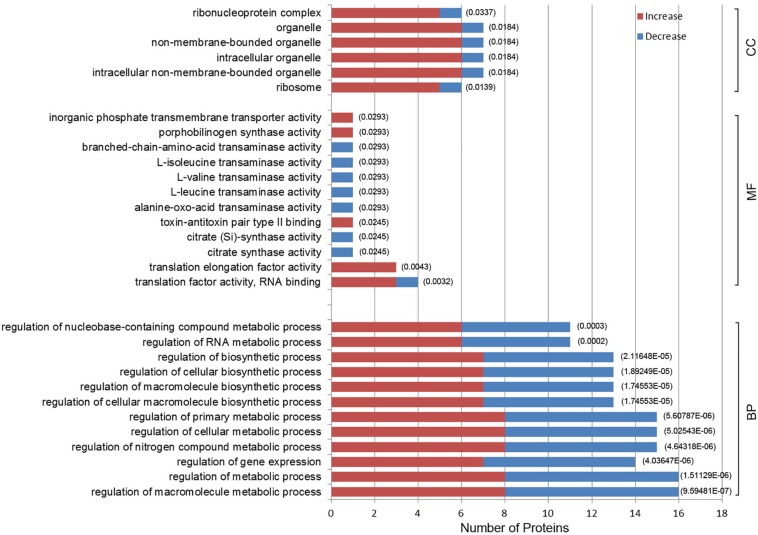
Gene Ontology (GO) enrichment analysis of 96 differentially expressed proteins in *N. gonorrhoeae* isolates. The histogram shows the top-10 most enriched categories. The *P*-values are marked at the top of the bars. Complete list of the enriched proteins are provided in Supplementary Data Sheet S2. BP, biological process; MF, molecule function; CC, cellular component.

Gene ontology enrichment was also performed to determine functional properties of the differentially expressed proteins. Among the 96 differentially expressed proteins, 45 were linked to “biological process” (BP), 26 to “molecule function” (MF), and 6 to the “cellular component” (CC) (Figure 4 and Supplementary Data Sheet S2). The differentially expressed proteins were correlated with regulation of metabolic processes, gene expression and biosynthetic processes. Most of the differentially expressed proteins have predicted functions associated with translation factor activity, RNA binding, translation, and transaminase activity. The enriched GO terms for the CC category included the ribosome, organelles and the ribonucleoprotein complex (Figure 4). A0A0M3GY07 (elongation factor Ts, EF-Ts) and A0A1D3FQV3 (translation initiation factor, InfB) were the highest frequency proteins in CC. Moreover, A0A0M3GXZ1 (conjugal transfer protein, TraJ), A0A0M3GY07 (EF-Ts), and A0A0M3GY33 (peptide transporter) were the highest frequency proteins for the differentially expressed proteins in the BP category.

### Three Amino Acid Metabolic Pathways Are Enriched and Three Network Subclusters Are Predicted by PPI Analysis

Kyoto encyclopedia of genes and genomes (KEGG) pathway analysis was used to examine pathways of the differentially expressed proteins. The 3 pathways included 2-Oxocarboxylic acid metabolism (ngk01210) and valine, leucine and isoleucine biosynthesis (ngk00290) and degradation (ngk00280) (*P* < 0.05) (Table 3). Six differentially expressed proteins (Q5F8G6, Q5F873, D6H9Y3, A0A1D3IB26, A0A1D3FF29, and D6HBH4) were involved in the three enrichment pathways, and all of the 6 proteins were downregulated. The function of Q5F873 (citrate synthase) was associated with energy metabolism. The function of D6HBH4 (3-hydroxyacid dehydrogenase), A0A1D3IB26 (Aminotransferase), D6H9Y3 (Aspartate-semialdehyde dehydrogenase) and Q5F8G6 (Didhydroxy-acid dehydratase) were associated with amino acid metabolism.

**TABLE 3:** Three enriched pathways of 96 differentially expressed proteins.

**Pathway description**	**Pathway ID**	***P* value**	**Matching proteins**
2-Oxocarboxylic acid metabolism	ngk01210	0.00152	Q5F8G6, Q5F873, D6H9Y3, A0A1D3IB26, A0A1D3FF29
Valine, leucine and isoleucine biosynthesis	ngk00290	0.012548	Q5F8G6, A0A1D3IB26, A0A1D3FF29
Valine, leucine and isoleucine degradation	ngk00280	0.018554	D6HBH4, A0A1D3FF29

**FIGURE 5:**
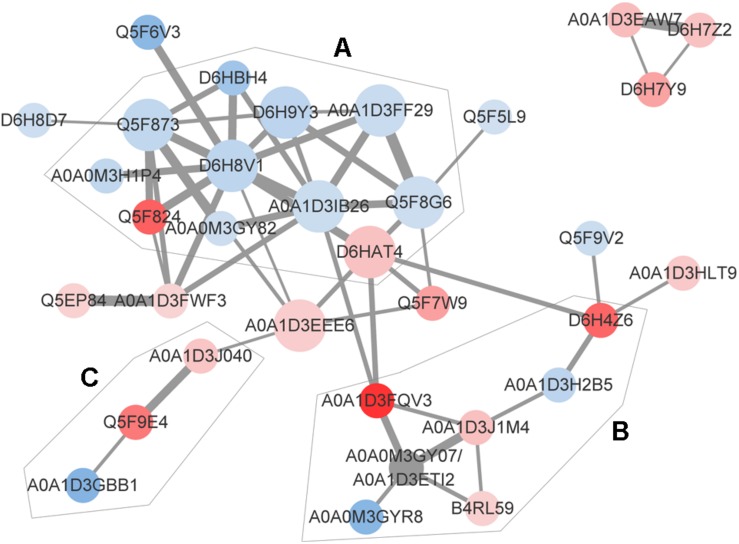
Predicted protein-association network. Graphic representations of predicted protein-protein interactions were generated with the STRING database and cytoscape software version 3.7. Nodes represent proteins. Red and blue colors indicate increases and decreases in protein expression, respectively. The depth of color represents the degree and the size represents the *P*-value of differential expressed proteins. The edge lines between nodes represent the biological relationship, and the width of lines represents the protein interaction intensity. Based on the biological function of these proteins, 3 enriched clusters were identified: **(A)** Metabolic process, **(B)** transcription and translation, and **(C)** bacterial virulence. The UniProt identity of the proteins are shown.

The identification and intensity of the protein interactions were predicted by PPI analysis. Approximately 34% (33/96) of the differentially expressed proteins were present in the three predicted protein network subclusters (Figure 5). Eleven differentially expressed proteins were present in the metabolic pathways, and 81.81% (9/11) of which were downregulated. In addition, the 6 proteins involved in the 3 enriched pathways were also present in the metabolic interaction network and acted as the hubs of the metabolic subcluster (Figure 5, Cluster A). Proteins of the second cluster were involved in the structural constituents of the ribosome and rRNA binding (Figure 5, Cluster B). This cluster included 30S ribosomal protein S6 (A0A1D3J1M4), 50S ribosomal protein L31 type B (B4RL59), the translation initiation factor InfB (A0A1D3FQV3) and ribonuclease R (A0A1D3H2B5). The two ribosomal proteins and InfB were upregulated while the ribonuclease R was downregulated. The third network predicted was related with 3 pilus proteins (Figure 5, Cluster C). Two pilus assembly proteins (Tfp pilus assembly protein FimT A0A1D3J040 and Tfp pilus assembly protein PilW Q5F9E4) were upregulated, while PilC2 (Tfp pilus assembly protein, tip-associated adhesin PilY1 A0A1D3GBB1) was downregulated. Seven proteins with an above-average degree of connectivity (>3.33) were defined as hub genes comprising D6H8V1, A0A1D3IB26, Q5F873, D6HAT4, Q5F8G6, A0A1D3EEE6 and D6H9Y3 (Table 4). It was predicted that protein succinate semialdehyde dehydrogenase (D6H8V1) interacts with 4 up- and 7 down-regulated proteins.

**TABLE 4:** Seven hub proteins selected from PPI network.

**UniProt entry**	**Protein description**	**FC**	**Degree**	**Betweenness centrality**	**Closeness centrality**	**Eccentricity**	**Stress**
D6H8V1	Succinate semialdehyde dehydrogenase	−2.28	11	0.28	0.48	4	414
A0A1D3IB26	Aminotransferase	−1.74	9	0.21	0.46	5	324
Q5F873	Citrate synthase	−2.13	9	0.14	0.44	4	212
D6HAT4	Riboflavin biosynthesis protein RibD	2.6	6	0.32	0.48	4	420
Q5F8G6	Dihydroxy-acid dehydratase	−1.73	6	0.10	0.40	5	162
A0A1D3EEE6	Superoxide dismutase	2.05	5	0.22	0.45	4	266
D6H9Y3	Aspartate-semialdehyde dehydrogenase	−2.31	5	0.012	0.40	5	32

*PPI, protein-protein interactions; FC, fold change.*

### Verification of 28 Differentially Expressed Proteins at the RNA Level

We performed qRT-PCR to confirm the RNA expression level of the differentially expressed proteins. The results verified 22 out of the 28 selected proteins (Table 5 and Supplementary Figure S1). Accordingly, 11 out of 15 downregulated proteins and 11 out of 13 upregulated proteins were in agreement at the protein and RNA levels as determined by iTRAQ and qRT-PCR (Table 5). Seven proteins (A0A1D3IYI7, A0A1P8DWD0, Q5F6C9, A0A171IPV7, A0A171IPV8, A0A1D3FJ50 and A0A1D3FPX7) showed differences in fold changes, but the directions of regulation demonstrated at the RNA levels or protein levels were consistent (Supplementary Table S2).

**TABLE 5:** Congruence of iTRAQ and qRT-PCR results on 28 differentially expressed proteins.

**UniProt entry**	**Gene name**	**Protein description**	**FC (iTRAQ)**	**FC (qRT-PCR)**
A0A1D3ETI2	*tsf*	Elongation factor Ts	−6.41	2.4
A0A1D3F5Y3	*dmlR_2*	LysR family transcriptional regulator	−1.65	−1^NS^
A0A1D3FVE4	*ssuB*	ABC transporter ATP-binding protein	−2.34	−1.51^a^
A0A1D3GBB1	*WHOM_00054C*	PilC2	−4.45	−1.58^NS^
A0A1D3IB26	*NCTC10931_02268*	Aminotransferase	−1.74	−1.57^a^
B4RK88	*NGK_0548*	Putative oxidoreductase, NAD(P)H-flavin	−7.12	−1.09^NS^
B4RQR4	*NGK_2270*	AdhesinMafA	−5.37	−1.65^a^
D6H9Y3	*asd*	Aspartate-semialdehyde dehydrogenase	−2.31	−4.35^a^
D6HBH4	*NGMG_02139*	3-hydroxyacid dehydrogenase	−3.35	−1.84^a^
Q5F6V3	*adhP*	Ethanol-active dehydrogenase/acetaldehyde-active reductase	−4.18	−2.31^a^
Q5F873	*gltA*	Citrate synthase	−2.13	−1.98^a^
Q5F8G6	*ilvD*	Dihydroxy-acid dehydratase	−1.73	−3.07^a^
A0A0M3GY07	*tsf*	Elongation factor Ts, EF-Ts	3.75	2.39^a^
A0A1D3E8W0	*WHOL_00301*	Membrane protein	2.03	5.53^a^
A0A1D3EEE6	*sodB*	Superoxide dismutase	2.05	1.66^a^
A0A1D3FQV3	*infB*	Translation initiation factor IF-2	9.63	1.44^NS^
A0A1D3G8M1	*WHOG_00181C*	ABC transporter substrate-binding protein	1.85	3.89^a^
A0A1D3J040	*fimT*	Fimbrial protein FimT	2.46	3.37^a^
D6H4Z6	*NGMG_01890*	16S RNA methyltransferase	7.13	2.99^a^
Q5F824	*ackA*	Acetate kinase	7.44	1.52^NS^
Q5F9E4	*NGO_0454*	Pilus assembly protein PilW	6.2	1.65^a^
A0A1D3IYI7	*WHOF_00049C*	Uncharacterized protein	−9.7	Downregulated^a^*
A0A1P8DWD0	*mtrR*	MtrR	−2.96	Downregulated^a^*
Q5F6C9	*NGO_1630*	Repressor	−6.95	Downregulated^a^
A0A171IPV7	*NGTW08_p0042*	TraM	3.66	Upregulated^a^*
A0A171IPV8	*NGTW08_p0043*	TraL	2.87	Upregulated^a^*
A0A1D3FJ50	*WHOL_PCO00023*	Conjugal transfer protein TrbF	3.5	Upregulated^a^*
A0A1D3FPX7	*vapD_1*	VapD	8.62	Upregulated^a^*

FC, fold change; NS, no statistically significant; a, congruence; *Proteins are shown as “Upregulated” or “Downregulated” instead of FC values, due to the highly elevated ΔΔCt and the cycle threshold (Ct) value, as shown in Supplementary Table S2.

## Discussion

To our knowledge, this is the first study to develop a proteomic profile for ESC-resistant clinical *N. gonorrhoeae* isolates using iTRAQ technology and nano-HPLC-MS/MS analysis. We identified 96 differently expressed proteins among the three ESC-resistant clinical *N. gonorrhoeae* isolates as compared to the *N. gonorrhoeae* ATCC49226 strain. The proteomic profiles of ESC-resistant *N. gonorrhoeae* isolates exhibited lower basal metabolism and enhanced capacities for protein synthesis. Differentially expressed proteins are associated with membrane permeability, transport, and transformation.

### Proteins Involved in Permeability of Cell Membranes

Expression of proteins related to cell membrane structure differed significantly between the three ESC-resistant *N. gonorrhoeae* isolates and the ESC-susceptible reference strain. Two proteins with predicted membrane localizations (MtrR and A0A1D3E8W0) were differentially expressed. MtrR is known to suppress the functions of the MtrCDE efflux pump, which is responsible for cellular expulsion of antimicrobials in *N. gonorrhoeae* (Lewis, 2014; Unemo and Shafer, 2014). In our study, we observed that the expression of MtrR in the ESC-resistant isolates were downregulated, suggesting decreased repression of the MtrCDE efflux pump, thereby increasing cellular expulsion of antimicrobials. This is consistent with findings that decreased expression of MtrR is associated with increased CRO MICs (El-Rami et al., 2019). Expression of a predicted membrane protein (A0A1D3E8W0) was upregulated in the three ESC-resistant isolates. However, the expression of this protein was downregulated in the WHO-L *N. gonorrhoeae* strain that was ESC-resistant (El-Rami et al., 2019). In El-Rami’s study, proteomic analysis was performed on proteins of subcellular fractionation, whereas in the present study, proteomic analysis was conducted on proteins from the whole cell extracts. Thus, the localinzation and the role of protein A0A1D3E8W0 in ESC-resistance in *N. gonorrhoeae* requires further study.

### Proteins Associated With Energy Metabolism, Transportation, and Metabolism of Amino Acids, Carbohydrates and Lipids

Cluster of orthologous groups analysis and GO enrichment analysis revealed that differentially expressed proteins are involved in the energy metabolism, transportation, and metabolism of amino acids, carbohydrates and lipids. The expression of dihydroxy-acid dehydratase IlvD (Q5F8G6), which is involved in carbohydrate transport and metabolism, was downregulated, which is consistent with findings from other reports (Zhao et al., 2019). IlvD has been shown to enhance the antioxidant capacity of *N. gonorrhoeae* by regulating the effects of the ABC transporter, MntABC via PerR (Wu et al., 2006). AckA (Q5F824), a putative acetate kinase, was upregulated in the three ESC-resistant isolates. AckA is involved in energy production and conversion. A previous study by others found that the expression of AckA was downregulated in ESC-resistant isolates when they were under ESC stressful conditions (Nabu et al., 2017). Amino acid metabolism influences the killing of antibiotic-resistant bacteria by enhancing antibiotic uptake and promoting ROS production (Lebeaux et al., 2014; Duan et al., 2016; Ye et al., 2018). We found that six proteins, including dihydroxy-acid dehydratase (Q5F8G6), citrate synthase (Q5F873), aspartate-semialdehyde dehydrogenase (D6H9Y3), aminotransferase (A0A1D3IB26), 3-hydroxyacid dehydrogenase (D6HBH4) and a protein of unknown function (A0A1D3FF29) were downregulated. Moreover, Q5F8G6, Q5F873, D6H9Y3 and A0A1D3IB26 act as core proteins in the metabolic interaction network. The downregulation in the expression of these enzymes in the ESC-resistant isolates suggests that decreased amino acid metabolism may be involved in ESC-resistance in *N. gonorrhoeae*.

### Proteins Involved in Transcription and Translation

We found that the expression of six proteins with predicted functions associated with translation, ribosomal structure, and biogenesis was differentially regulated in the three ESC-resistant *N. gonorrhoeae* isolates. These 6 proteins are elongation factor EF-Ts (A0A0M3GY07), 50S ribosomal protein L31 type B RpmE2 (B4RL59), 30S ribosomal protein S6 RpsF (A0A1D3J1M4) translation initiation factor InfB (A0A1D3FQV3), and ribonuclease R VacB (A0A1D3H2B5). RpsF and RpmE2 were upregulated. RpsF is present in many bacterial species, and genes of *rps*F and *rps*R are co-transcribed from the S6 operon in *Bacillus subtilis* (Fu et al., 2014). The gene silencing of *rps*F decreases the gene transcription level of *rps*R in the same operon and leads to *Escherichia coli* growth inhibition (Tang et al., 2014). VacB was downregulated in the 3 ESC-resistant isolates. VacB is involved in mRNA decay, which greatly influences final protein production (Cheng and Deutscher, 2005; Domingues et al., 2015). VacB co-migrates with single 30S ribosomal subunits and is involved in rRNA quality control process (Malecki et al., 2014). InfB was upregulated in the three ESC-resistant isolates. InfB controls the fidelity of translation initiation by selectively increasing the rate of 50S ribosomal subunit joining to 30S initiation complexes (ICs) that carry an N-formyl-methionyl-tRNA (Caserta et al., 2006; Caban et al., 2017). We found that elongation factor EF-Ts (Nyborg and Kjeldgaard, 1996) was upregulated. However, other reports established that the expression of elongation factor Tsf is downregulated in response to CRO induction in *N. gonorrhoeae* (Nabu et al., 2017). In the Nabu study, a sublethal concentration of CRO was included in the culture of *N. gonorrhoeae* ATCC49226 to prepare protein extracts. The stress of CRO may induce transient alterations in protein expression.

### Proteins Involved in the Type IV Secretion Systems

Five of the upregulated proteins in the 3 ESC-resistant isolates were part of the Type IV secretory systems (T4SSs). These upregulated proteins were VirB10 component TrbI (A0A171IPU0), VirB4 component TrbE (A0A171IPU4), VirD4 component/TraG/TraD family ATPase (A0A1D3FK04), VirB9 components TrbG (A0A171IPU2) and conjugal transfer protein TrbF (A0A1D3FJ50). T4SS proteins are involved in the translocation and spread of antibiotic resistance genes among bacterial cells (Ramsey et al., 2011; Christie et al., 2014; Guiney, 2017). VirD4 binds to the type IV coupling protein (T4CP). VirB4 is a component of the inner membrane complex that is involved in substrate transfer across the inner membrane. The outer membrane-associated lipoprotein VirB9 and the cell-envelope-spanning subunit VirB10 compose the core complex. VirB10 plays a central role in signal perception (Christie et al., 2014). These findings suggest the involvement of T4SS in the mechanisms underlying ESC-resistance in *N. gonorrhoeae*.

### Proteins Associated With ABC Transporters

Bacterial ABC transporters are involved in the uptake of nutrients, the extrusion of building blocks and drug resistance (Seeger and van Veen, 2009; Cuthbertson et al., 2010; Locher, 2016). Two proteins associated with ABC transporters were found to be differentially regulated in the three ESC-resistant *N. gonorrhoeae* isolates. An ABC transporter substrate-binding protein (A0A1D3G8M1) was upregulated, and this protein is essential for and specific to nutrient intake (Maqbool et al., 2015). The ABC transporter ATP-binding protein SsuB (A0A1D3FVE4) was downregulaed. Other studies also found that ABC transporters are differentially regulated in ESC-resistant *N. gonorrhoeae* isolates under ESC stressful conditions (Gong et al., 2016; Nabu et al., 2017). The results of our study and studies by others support that ABC transporters are involved in ESC-resistance in *N. gonorrhoeae*.

### Limitations

Pérez-Llarena and Bou (2016) reviewed proteomic analysis of AMR and categorized them into two categories, i.e., comparison of resistant and susceptible strains and bacterial responses to the presence of sub-MIC or sub-lethal concentrations of antimicrobials. Our study compared expression profiles of clinically resistant isolates and a laboratory susceptible strain. The use of laboratory susceptible strain may lead to bias as a laboratory strain may lose factors involving pathogenesis and fitness after many years storage and numerous subculture cycles. Comparison of clinical isolates with various MIC levels would be superior to using laboratory reference strains. Another factor that may bias the findings is the use of bacterial cells from plate-cultivation for 20 h (Nabu et al., 2014, 2017). Furthermore, proteomic analysis of bacteria in responses to antimicrobials or other stressful environments would have strengthened this study’s ability to determine whether the changes in expression observed in the ESC-resistant isolates reflected compromised fitness. In general, multivariable approaches combining both types of study designs would contribute to a better undersdanding of antimicrobial resistant mechanisms (Pérez-Llarena and Bou, 2016). The number of proteins identified in this study accounted for approximately 72% of protein-coding genes in *N. gonorrhoeae*, probably due to incomplete labeling of peptides and inability to detect low abundant proteins (Park et al., 2016; Pérez-Llarena and Bou, 2016). Our study does not reveal the extracellular mechanisms and the mechanisms caused by post-translational modifications. The findings of this study will need to be confirmed by gene inactivation and replacement technology and animal models (Chen et al., 2019).

## Conclusion

In conclusion, using iTRAQ comparative proteomics, we identified 96 differentially expressed proteins in three ESC-resistant *N. gonorrhoeae* clinical isolates. Forty of the 96 proteins were downregulated while 56 were upregulated. The mechanisms of ESC-resistance in *N. gonorrhoeae* are associated with membrane permeability, Type IV secretion, metabolism, and transportation of various substrates. To our knowledge, this is the first report of comparative proteomic analysis using naturally occurring ESC-resistant clinical *N. gonorrhoeae* strains. Further studies to biologically verify the involvement of the differentially expressed proteins in ESC-resistance in *N. gonorrhoeae* will be required. The present study provides insights into the mechanism underlying ESC-resistance in *N. gonorrhoeae* and valuable information for further studies aiming at potential antimicrobial targets.

## Data Availability Statement

The proteomic data generated for this study has been deposited to the PRIDE Archive (Perez-Riverol et al., 2019) under accession number PXD015296 (http://www.ebi.ac.uk/pride/archive/projects/PXD015296).

## Author Contributions

WG designed the study, collected and analyzed the data, and prepared the manuscript. ND and GY did the experiments, data analysis, and prepared the manuscript. YY designed the study and the experiments. YD and YW analyzed the data and prepared the manuscript.

## Conflict of Interest

The authors declare that the research was conducted in the absence of any commercial or financial relationships that could be construed as a potential conflict of interest.

## Footnotes

1 www.ncbi.nlm.nih.gov/COG/

2 http://kobas.cbi.pku.edu.cn/

## Supplementary Material

FIGURE S1 qRT-PCR analysis for the selected 28 differentially expressed proteins. ΔΔCt was used as “Fold Change”. NS, not statistically significant; **p* < 0.05, ***p* < 0.01, ****p* < 0.001 compared with *N. gonorrhoeae* ATCC 49226.

TABLE S1 Primers used in qRT-PCR.

TABLE S2 Ct value of identified proteins with highly elevated ΔΔCt.

DATA SHEET S1 The information of the quantifiable peptides and proteins.

DATA SHEET S2 The enriched terms for biological process, molecule function and cellular component.
