# Supplementary material for: Comparative Proteomics of Extended-Spectrum Cephalosporin-Resistant Neisseria gonorrhoeae Isolates Demonstrates Altered Protein Synthesis, Metabolism, Substance Transport, and Membrane Permeability
Source: Front Microbiol. 2020 Feb 19;11:169. doi: 10.3389/fmicb.2020.00169 (PMC7042406; doi:10.3389/fmicb.2020.00169)
Supplement: TABLE S1 — Primers used in qRT-PCR. [file Table_1.docx]

**Table S1.** Primers used in qRT-PCR

| UniproEntry | Gene name | Primer name | Size(bp) | Nucleotide sequence (5’-3’) |
| --- | --- | --- | --- | --- |
| A0A1D3ETI2 | tsf | tsf -F  tsf -R | 155 | AGAACGCCACATCTACACCGA  ATTGGGCAACAGTTTGGTCA |
| A0A1D3F5Y3 | dmlR_2 | dmlR_2-F  dmlR_2-R | 209 | CGCCACGTCGATTTGATT  TTCTGCTGGTTGGTGTAGGTC |
| A0A1D3FVE4 | ssuB | ssuB-F  ssuB-R | 222 | TTATGGACAACCCAGACGAAG  CCACCAGCATAGCAACCAA |
| A0A1D3GBB1 | WHOM_00054C | WHOM_00054C-F  WHOM_00054C-R | 173 | GATGTATTCCGCTTCGGTTTC  TCACGGTCTGCCTTGAGTTG |
| A0A1D3IB26 | NCTC10931_02268 | NCTC10931_02268-F  NCTC10931_02268-R | 98 | TTCAGTTGGAAGAGGAGGGC  GACATCGACTAAGATTTCATCAGGT |
| A0A1D3IYI7 | WHOF_00049C | WHOF_00049C-F  WHOF_00049C-R | 124 | TAATGGCGGACAATGCTATCT  TGACCGATAAACTTCCTCTGC |
| A0A1P8DWD0 | mtrR | mtrR -F  mtrR -R | 165 | CAACGAAATCGCCCAAAC  AATACCGTCCAAGAACCTCCT |
| B4RK88 | NGK_0548 | NGK_0548-F  NGK_0548-R | 220 | GCAAAGAGCAGGTTCTATCCG  TCCAAAGCATCCGCCATAC |
| B4RQR4 | NGK_2270 | NGK_2270-F  NGK_2270-R | 112 | CAATCAGACGGTAGCGGAAGT  GGGAAAGAAAGGCAGTGTCG |
| D6H9Y3 | asd | asd-F  asd-R | 134 | AACGACTTCGCCCACATTC  ATGTCCATTTTCGCCAGCT |
| D6HBH4 | NGMG_02139 | NGMG_02139-F  NGMG_02139-R | 190 | TGGGTAGGCTTAGGGCAAAT  CATAGTCGGAAACCATCAGGAA |
| Q5F6C9 | NGO_1630 | NGO_1630-F  NGO_1630-R | 148 | ACCAACACCATCACCCTCAAC  CGTTTGTCAGATTGTTTGTCCC |
| Q5F6V3 | adhP | adhP-F  adhP-R | 141 | CGACATCAACGACGACAAGC  GCAGATACGGCGGTTACGA |
| Q5F873 | gltA | gltA-F  gltA-R | 145 | GCAAACTGTATCCCAATGTCG  GAAGGATCGCTAATCATCTCGT |
| Q5F8G6 | ilvD | ilvD-F  ilvD-R | 146 | GCCGTTTGGACTTGATTGAC  GGTCAGGCAGTTCATCGAGT |
| A0A0M3GY07 | tsf | tsf-F  tsf-R | 101 | AGCATTCGTGATGAACCCTG  CGATACCGTCGCCTACTTTG |
| A0A171IPV7 | NGTW08_p0042 | NGTW08_p0042-F  NGTW08_p0042-R | 81 | AAGGAAACTCGGAACACGG  TTTGAACGCCAAGATAATGACT |
| A0A171IPV8 | NGTW08_p0043 | NGTW08_p0043-F  NGTW08_p0043-R | 130 | TGCGTTTATTCCGCTTTGT  TCCGTTGACCGTATGCTTC |
| A0A1D3E8W0 | WHOL_00301 | WHOL_00301-F  WHOL_00301-R | 147 | TTGTTTGGACATCCCGCC  GGGTAATCGCATCTTTCTGGT |
| A0A1D3EEE6 | sodB | sodB-F  sodB-R | 164 | ACGGCAAACACCATCAAACC  CGTGAAACCCAGCCAGTAGAA |
| A0A1D3FJ50 | WHOL_PCO00023 | WHOL_PCO00023-F  WHOL_PCO00023-R | 156 | TGCTGAATCCGAAAGACGC  CCCAGTACACCTGCCAAGAGT |
| A0A1D3FPX7 | vapD_1 | vapD_1-F  vapD_1-R | 142 | ACCATCAGCAAGGCAGTGTAT  ATTTCCTCAATCCGCAACAT |
| A0A1D3FQV3 | infB | infB-F  infB-R | 212 | CATCTCCGTTTCCAAGGTCG  CTTCCATGATTTCGTTGTAGCC |
| A0A1D3G8M1 | WHOG_00181C | WHOG_00181C-F  WHOG_00181C-R | 178 | CGTCAAACAGAGCAAAGTATCCT  GGCATAGTCGGACATAAGAACA |
| A0A1D3J040 | fimT | fimT-F  fimT-R | 111 | CAACCTTTTGCGTTTCTCCA  CCGAAGTCGCATTGGTTGT |
| D6H4Z6 | NGMG_01890 | NGMG_01890-F  NGMG_01890-R | 135 | CCGAAACACTGGCACAACA  CAACGCCTTAGCCGTGATAC |
| Q5F824 | ackA | ackA-F  ackA-R | 241 | TACAAATACGCCGTTCCGC  TACCCATTACCAGCCCTTCC |
| Q5F9E4 | NGO_0454 | NGO_0454-F  NGO_0454-R | 147 | AGGGCGGTTATGATGGTATG  GAATGGCAAGACGCTCGTT |
| A0A1D3FJC8 | WHOG_00311 | WHOG_00311-F  WHOG_00311-R | 219 | CCAAGCAATCAATCTTACCGA  TCGTAGACATCTATCCCCCTTAA |
| - | 16S rRNA | 16S rRNA-F  16S rRNA-R | 182 | GCGGAGCCAATCTCACAA  TGCGGTTACC CTACCTACTTCT |
