# Supplementary material for: Comparative Proteomics of Extended-Spectrum Cephalosporin-Resistant Neisseria gonorrhoeae Isolates Demonstrates Altered Protein Synthesis, Metabolism, Substance Transport, and Membrane Permeability
Source: Front Microbiol. 2020 Feb 19;11:169. doi: 10.3389/fmicb.2020.00169 (PMC7042406; doi:10.3389/fmicb.2020.00169)
Supplement: TABLE S2 — Ct value of identified proteins with highly elevated ΔΔCt. [file Table_2.docx]

**Table S2.** Ct value of identified proteins with highly elevated △△Ct.

|  | 16S rRNA | A0A1D3IYI7 | A0A1P8DWD0 | Q5F6C9 | A0A171IPV7 | A0A171IPV8 | A0A1D3FJ50 | A0A1D3FPX7 |
| --- | --- | --- | --- | --- | --- | --- | --- | --- |
| SH40 | 13.39 | 34.80 | 37.74 | 36.17 | 19.96 | 18.97 | 20.57 | 16.70 |
| SH41 | 13.07 | 35.68 | 37.70 | 40.21 | 19.62 | 18.38 | 20.12 | 16.45 |
| SH48 | 12.97 | 36.32 | 37.63 | 39.48 | 19.58 | 18.48 | 20.19 | 16.62 |
| ATCC49226 | 12.72 | 20.11 | 26.30 | 23.25 | 36.85 | 34.98 | 36.36 | 33.56 |
